# Supplementary material for: ATP128 Clinical Therapeutic Cancer Vaccine Activates NF-κB and IRF3 Pathways through TLR4 and TLR2 in Human Monocytes and Dendritic Cells
Source: Cancers (Basel). 2022 Oct 20;14(20):5134. doi: 10.3390/cancers14205134 (PMC9600632; doi:10.3390/cancers14205134)
Supplement: Supplementary file 1 [file cancers-14-05134-s001.zip › cancers-1919637-supplementary figures and tables.pdf]

Figure S1

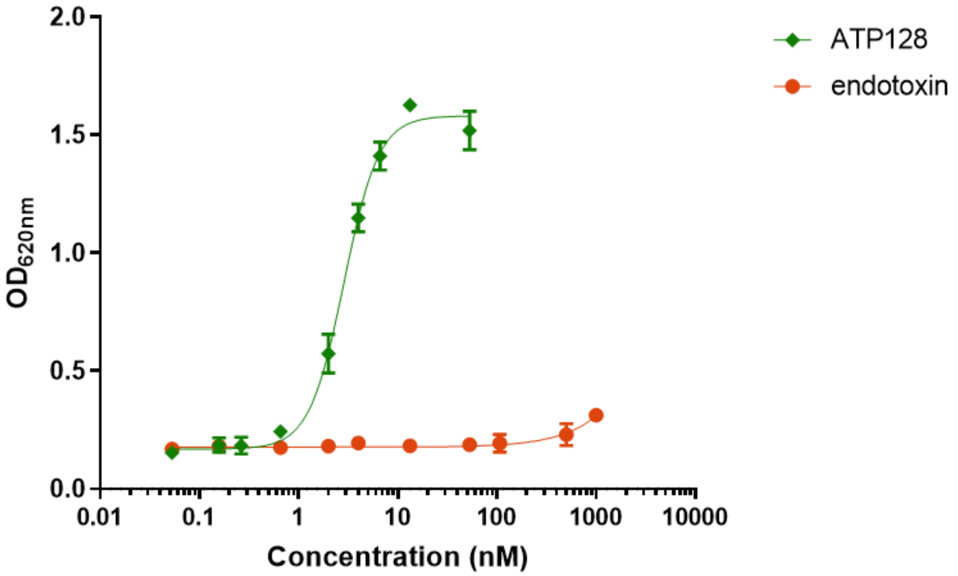

Figure S1. Endotoxin level comparable to the one present in the constructs do not activate NF-κB pathway. THP-1 MD2-CD14 cells were incubated with increasing concentrations of endotoxin. ATP128 vaccine was used as a positive control. After 18 hours, supernatant was recovered, and SEAP activity was measured by QUANTI-Blue assay.

Table S1

Table S1 **Endotoxin levels in ATP128 variants**

**Protein**	**Endotoxin level**
ATP128 FL	<0.3 EU/mg
ATP128 w/o Anaxa	1.15 EU/mg
ATP128 w/o CPP	2.49 EU/mg

FL, full length

Endotoxin levels of ATP128, ATP128 w/o Anaxa and ATP128 w/o CPP proteins

Figure S2

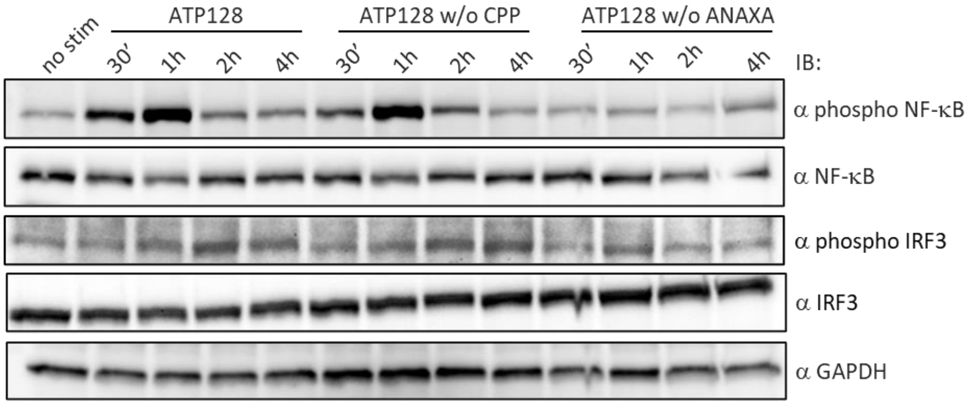

Figure S2. NF-κB and IRF3 phosphorylation induced by ATP128 constructs. THP-1 Dual cells were stimulated with 300 nM of various ATP128 constructs at different time points and phosphorylation of NF-κB and IRF3 was assessed through Western Blot using specific phospho-antibodies. Anti-GAPDH was used as normalizer.

Figure S3

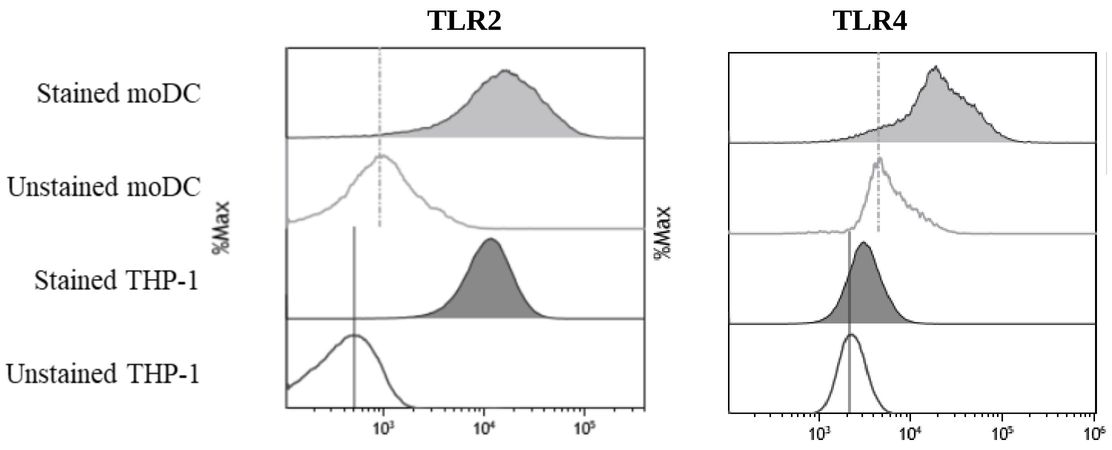

Figure S3. THP-1 cell line and human moDCs express different levels of TLR2 and TLR4. THP-1 cell line and human moDCs were assessed for the surface expression of TLR2 and TLR4.

Figure S4

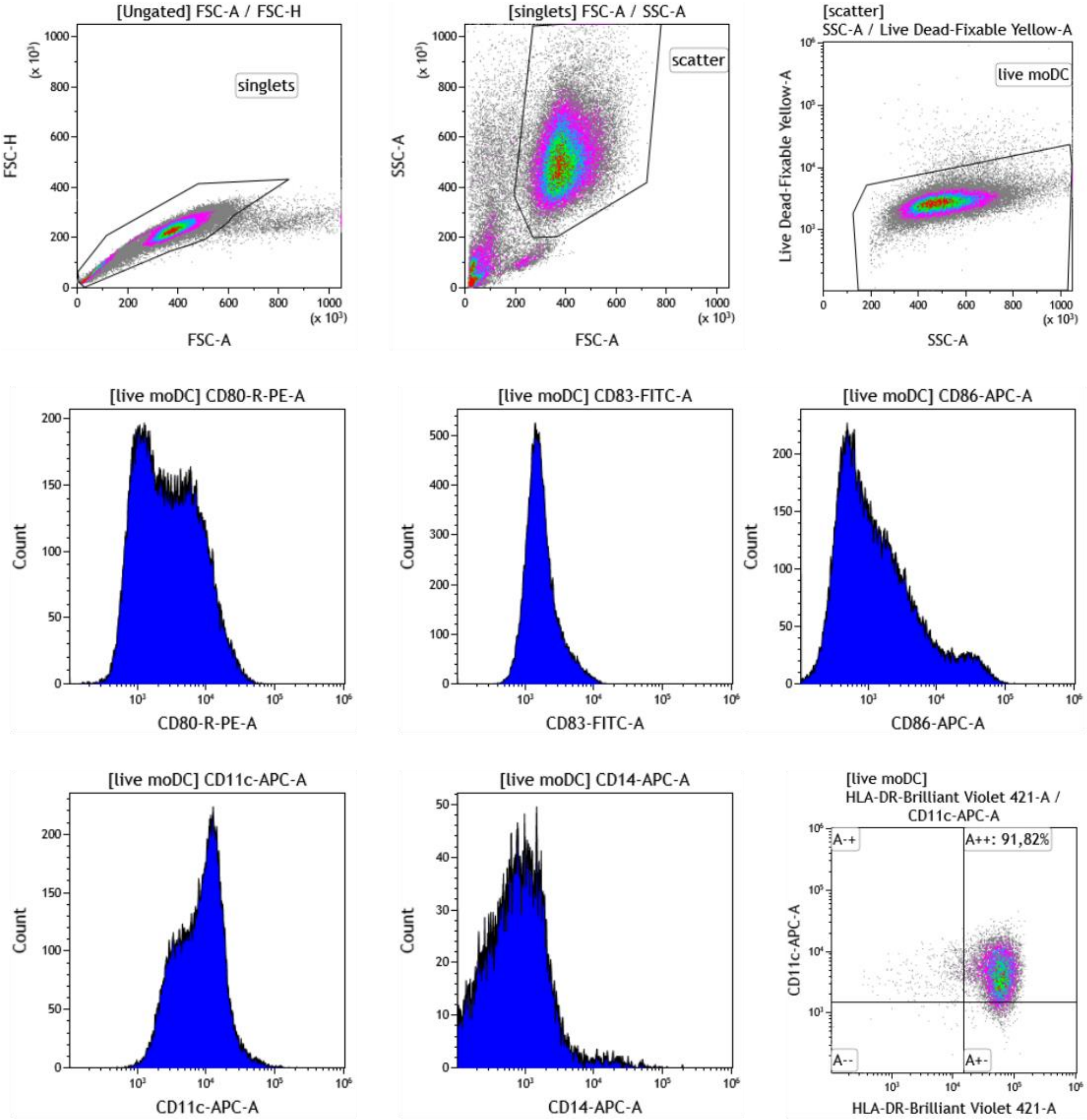

[Ungated] FSC-A / FSC-H
singlets
FSC-H
FSC-A
[singlets] FSC-A / SSC-A
scatter
SSC-A
FSC-A
[scatter]
SSC-A / Live Dead-Fixable Yellow-A
live moDC
Live Dead-Fixable Yellow-A
SSC-A
[live moDC] CD80-R-PE-A
Count
CD80-R-PE-A
[live moDC] CD83-FITC-A
Count
CD83-FITC-A
[live moDC] CD86-APC-A
Count
CD86-APC-A
[live moDC] CD11c-APC-A
Count
CD11c-APC-A
[live moDC] CD14-APC-A
Count
CD14-APC-A
[live moDC]
HLA-DR-Brilliant Violet 421-A /
CD11c-APC-A
A-+
A++: 91,82%
A--
A+-
CD11c-APC-A
HLA-DR-Brilliant Violet 421-A
